# Versatile Activated Carbon Fibers Derived from the Cotton Fibers Used as CO_2_ Solid-State Adsorbents and Electrode Materials

**DOI:** 10.3390/molecules29133153

**Published:** 2024-07-02

**Authors:** Peiyu Wang, Hang Liu, Wenting Zhu, Wanjun Chen, Xiangli Wang, Le Yang, Bao Yang, Qiong Chen, Cairang Limao, Zhuoma Cairang

**Affiliations:** Gansu Engineering Research Center for Eco-Environmental Intelligent Networking, Key Laboratory for Electronic Materials of Northwest Minzu University, College of Electric Engineering, Northwest Minzu University, Lanzhou 730030, China; 18353818980@163.com (H.L.); 17352112812@163.com (W.Z.); wjchen@xbmu.edu.cn (W.C.); phy_wang@163.com (X.W.); 184112732@xbmu.edu.cn (L.Y.); yangbcsu@163.com (B.Y.); 281041662@xbmu.edu.cn (Q.C.); limao@xbmu.edu.cn (C.L.); 272970673@xbmu.edu.cn (Z.C.)

**Keywords:** activated carbon fibers, cotton fibers, microporous structure, CO_2_ capture, electrochemical capacitance

## Abstract

Activated carbon has an excellent porous structure and is considered a promising adsorbent and electrode material. In this study, activated carbon fibers (ACFs) with abundant microporous structures, derived from natural cotton fibers, were successfully synthesized at a certain temperature in an Ar atmosphere and then activated with KOH. The obtained ACFs were characterized by field emission scanning electron microscopy (FESEM), transmission electron microscopy (TEM), elemental analysis, nitrogen and carbon dioxide adsorption–desorption analysis, X-ray diffraction (XRD), X-ray photoelectron spectroscopy (XPS) and N_2_ adsorption–desorption measurement. The obtained ACFs showed high porous qualities and had a surface area from 673 to 1597 m^2^/g and a pore volume from 0.33 to 0.79 cm^3^/g. The CO_2_ capture capacities of prepared ACFs were measured and the maximum capture capacity for CO_2_ up to 6.9 mmol/g or 4.6 mmol/g could be achieved at 0 °C or 25 °C and 1 standard atmospheric pressure (1 atm). Furthermore, the electrochemical capacitive properties of as-prepared ACFs in KOH aqueous electrolyte were also studied. It is important to note that the pore volume of the pores below 0.90 nm plays key roles to determine both the CO_2_ capture ability and the electrochemical capacitance. This study provides guidance for designing porous carbon materials with high CO_2_ capture capacity or excellent capacitance performance.

## 1. Introduction

Porous carbons have received attention due to their large surface area, abundant porosity, thermal and chemical stability, and high conductivity and other advantages. They have shown great application prospects in adsorption and separation [1,2,3], energy storage and conversion [4,5], catalysis, and other fields [6]. Among them, ACF samples are one of the most important types, with faster adsorption kinetics than particle types [7].

Biochar is usually produced by carbonizing the original biomass material and then activating it, which has been a recent research hotspot. So far, various biomass-derived activated carbons, macadamia nut shell [8], cow dung [9], pine nut shells [10], macadamia nut shell-based carbon [11], and so on, have been reported [12,13,14,15,16,17,18,19,20,21,22,23,24,25]. These activated carbons have shown promising applications as solid CO_2_ adsorbents [8,9,10,11,12,13,14,15,16,17,18,19,20,21,22,23,24,25,26,27] and electrode materials for supercapacitors [28,29,30,31,32,33,34,35,36,37,38,39].

In terms of structural adjustment and optimization of porous carbon materials, it is even more necessary to explore their underlying mechanisms in order to conduct research at a more microscopic level. In addition, with energy shortages and environmental pollution, it is necessary to develop simple, clean, and inexpensive porous carbon materials. This will help address energy and environmental crises and have significant impacts in a wider range of areas.

Everyone knows that the CO_2_ capture capacity of porous carbon is mainly determined by its porous structure. For example, some reports showed that the narrow micropores have a great contribution to the CO_2_ capture capability [40,41]. Thus, more and more studies devote to improve the CO_2_ capture ability by adjusting the porous structure of activated carbons [8,9,10,11,12,42].

In addition, developing clean energy as fossil fuels is an effective way to reduce carbon dioxide emissions [43]. At present, supercapacitors are an efficient energy storage device with advantages such as high power density, long cycle life, and environmental friendliness [5,44]. Electric double-layer supercapacitors (EDLCs) generate a layer at the electrode-electrolyte interface by generating dual charges. Therefore, their electrochemical performance is closely related to the pore structure. Therefore, porous carbon has shown great potential in the preparation of supercapacitors due to its multifunctional and adjustable pore structure [5,44,45].

Natural cotton fiber (CF) is a natural renewable agricultural resource and it has been widely used for many fields. CF consists of cellulose which is a natural polymer composed of many sugar glucose molecules. CF is also among the longest fiber types known in the plant kingdom. Each CF is basically a hollow tube a few centimeters in length. Although Xing et al. used a one-step activation method to prepare porous carbon derived from cotton fibers, the one-step method had a strong destructive effect on the fibers, making it difficult for the generated porous carbon material to maintain its fibrous shape [46]. Muhammad et al. prepared spider silk-derived ACFs for high-pressure CO_2_ capture [26]. We also used silk fibers as raw materials to prepare nitrogen-doped ACFs for CO_2_ capture [27].

In this study, CF was used as a crude material to prepare high-performance ACF by simple carbonization in an Ar atmosphere and then activated with KOH. The long hollow CF provided an important morphological foundation for the excellent CO_2_ capture capacity and high specific capacitance. The CO_2_ adsorption and electrochemical performance of the obtained ACFs were measured. It was found that the CO_2_ adsorption performance and electrochemical performance of the ACF material was relatively superior. We also characterized the structure of the ACF material in detail, and the effects of the porosity characteristics (pore volume) of the ACF material on the CO_2_ capture capacity and the specific capacitance were also systematically explored.

## 2. Results and Discussion

### 2.1. Sample Structure

**Figure 1:**
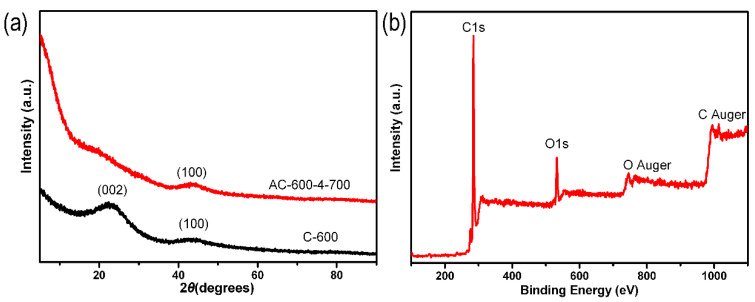
(**a**) XRD patterns of the C-600 and AC-600-4-700 samples. (**b**) XPS full-spectrum of the AC-600-4-700 sample.

A series of ACF samples were prepared by adjusting the carbonization temperature, weight ratio of KOH and C-x, and activation temperature. Figure 1a showed the XRD measurement characteristics of C-600 and AC-600-4-700. In Figure 1a, there were two diffraction peaks of the C-600 sample displayed at 23° and 43°, related to the (002) and (100) planes of amorphous graphite-like carbon. The intensity of the original (002) peak significantly decreased and almost disappeared, but the 100 peak still existed after activation (AC-600-4-700). The high diffraction intensity of AC-600-4-700 samples at low angle scattering indicated the presence of a large number of pores [47,48]. Using XPS measurement, 12.48 wt% O and 87.52 wt% C were detected in the AC-600-4-700 sample (Figure 1b).

**Figure 2:**
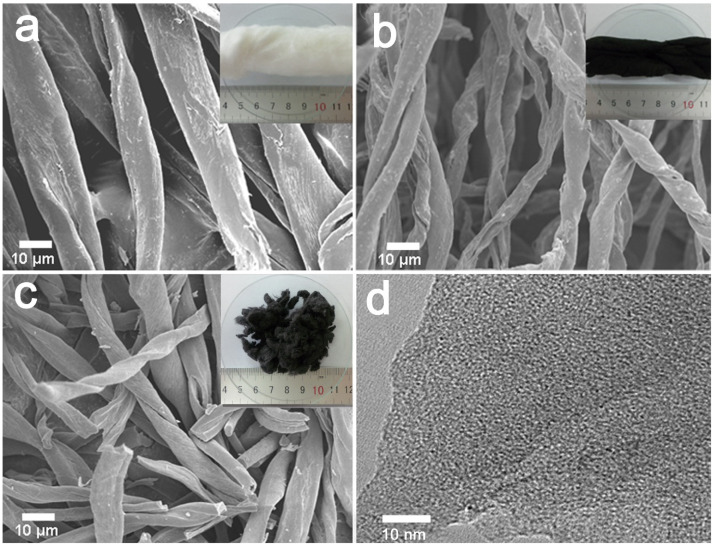
SEM images and the digital photograph (insert) of the materials derived from CFs: (**a**) natural CFs, (**b**) C-600, (**c**) AC-600-4-700, (**d**) TEM image of the AC-600-4-700 sample.

Figure 2 shows SEM images and digital photographs of the surface morphology of CF, C-600, and AC-600-4-700 derived from cotton fibers (inserted). The C-600 slightly shrank in size and shape after the carbonization (Figure 2a,b). Furthermore, KOH activation transformation led to local curvature and many defects generated on the surface of C-600 fibers, giving rise to micropores, which resulted in the cotton carbon fibers cracking into pieces, as shown in Figure 2c. The TEM image of AC-600-4-700 (Figure 2d) evidenced that the porosity was made up of randomly oriented uniform micropores.

**Figure 3:**
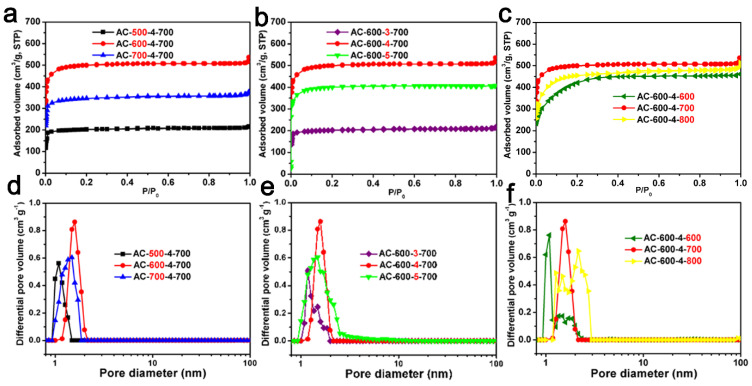
The N_2_ adsorption and desorption curves of ACF samples: (**a**) AC-x-4-700 (x = 500, 600 and 700); (**b**) AC-600-y-700 (y = 3, 4 and 5); (**c**) AC-600-4-z (z = 600, 700 and 800). The corresponding NLDFT pore size distribution of ACFs: (**d**) AC-x-4-700 (x = 500, 600 and 700); (**e**) AC-600-y-700 (y = 3, 4 and 5); (**f**) AC-600-4-z (z = 600, 700 and 800).

The N_2_ adsorption–desorption isotherms and corresponding pore size distribution of the ACF samples are shown in Figure 3. The Type IV isotherms of AC-500-4-700 and AC-600-4-800 were related to capillary condensation occurring in the mesopores and exhibit significant hysteresis loops, according to the IUPAC classification. The Type I isotherms of the remaining samples were obtained [49,50]. For most samples, the main N_2_ adsorption occurred at less than 0.1 P/P_0_. At higher P/P_0_ values, a relatively horizontal adsorption platform appeared, indicating that ACF samples prepared through carbonization and KOH activation were highly microporous, consistent with the TEM images [51].

As indicated in Table 1, these ACF samples have an SSA from 673 to 1597 m^2^/g, and a pore volume from 0.33 to 0.79 cm^3^/g. From Table 1, It can be seen that even under the same activation conditions (activated with KOH chemical etching agent at 700 °C for one hour), the SSA and pore volume of AC-x-4-700 (x = 500, 600, 700) samples are different and related to the carbonization temperature. In the Table 1, it can be seen that AC-600-4-700 had the highest SSA and pore volume among AC-600-y-700 (y = 3, 4, 5) materials, and it can also be seen that the AC-600-4-z (z = 600, 700, 800) materials exhibited relatively high surface areas and pore volumes compared to other series. The PSD curves of ACF samples are shown in Figure 3d–f. The curves show that the ACF samples were almost composed of micropores (<2 nm). The pore structure of biomass-derived ACFs is closely related to their own structure, for example, spider fiber carbon fibers and silk fibers have a relatively large SSA and pore volume (spider silk-derived ACF: 2730 m^2^/g and 1.56 cm^3^/g; silk fiber-derived ACF: 3000 m^2^/g and 1.38 cm^3^/g) [26,27]. However, relatively speaking, cotton fiber is cheaper and easier to obtain.

### 2.2. CO_2_ Capture Capacity

**Figure 4:**
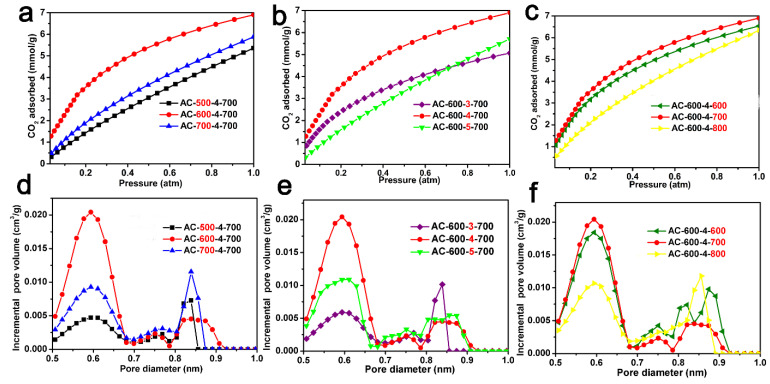
CO_2_ adsorption curves of ACF samples: (**a**) AC-x-4-700 (x = 500, 600 and 700); (**b**) AC-600-y-700 (y = 3, 4 and 5); (**c**) AC-600-4-z (z = 600, 700 and 800). The pore size distribution for ACFs derived from CO_2_ adsorption using the NLDFT model: (**d**) AC-x-4-700 (x = 500, 600 and 700); (**e**) AC-600-y-700 (y = 3, 4 and 5); (**f**) AC-600-4-z (z = 600, 700 and 800).

The CO_2_ adsorption isotherms (0 °C, 1.0 atm) and the pore size distribution of the ACF samples derived from CO_2_ adsorption data using the NLDFT model are shown in Figure 4, respectively. Table 1 also lists the CO_2_ absorption of the ACF samples (0 °C and 25 °C, 1.0 atm) and the pore characteristics calculated based on the CO_2_ adsorption isotherms. Figure 4a shows the CO_2_ adsorption isotherms of AC-x-4-700 (x = 500, 600, and 700), with a CO_2_ adsorption capacity ranging from 4.3 to 6.9 mmol/g. Figure 4b shows the CO_2_ absorption isotherms of AC-600-x-700 (x = 3, 4, and 5). These ACF samples exhibit high CO_2_ absorption in the range of 4.9–6.9 mmol/g. Figure 4c shows the CO_2_ adsorption isotherms of AC-600-4-z (z = 600, 700, and 800). AC-600-4-z materials had large CO_2_ uptakes in the range of 6.3–6.9 mmol/g. It is worth mentioning that AC-600-4-700 exhibited a significant optimal CO_2_ absorption of 6.9 mmol/g. This value is comparable to most other biomass-derived activated carbons (Table 2) [8,9,10,11,12,13,14,15,16,17,18,19,20,21,22,23,24,25,27]. In Figure 4d–f, the PSD of the ACF samples derived from CO_2_ adsorption data below 1.0 nm are displayed, indicating the presence of abundant micropores in the ACF materials.

**Figure 5:**
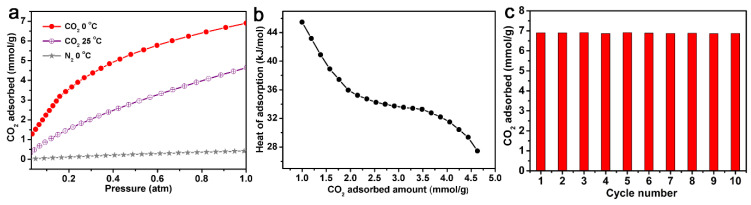
(**a**) CO_2_ and N_2_ adsorption curves of AC-600-4-700 at 0 and 25 °C, 1 atm. (**b**) Isosteric heats of CO_2_ adsorption on AC-600-4-700 calculated from the adsorption isotherms at 0 and 25 °C, 1 atm. (**c**) CO_2_ multi-circle adsorption curves of AC-600-4-700 at 0 °C.

The adsorption capacity of AC-600-4-700 for N_2_ was also measured at 0 °C and 1 atm. As shown in Figure 5a, compared with CO_2_ adsorption at the same condition (6.9 mmol/g), the N_2_ adsorption capacity could only reach a maximum of 0.4 mmol/g, with a much lower adsorption capacity. This meant that AC-600-4-700 was a selective adsorbent that could be applied for the separation of CO_2_ and N_2_. The adsorption capacity of AC-600-4-700 for CO_2_ at 25 °C and 1 atm could reach 4.6 mmol/g.

The isosteric heat of adsorption (Q_st_) calculated based on the CO_2_ adsorption isotherms of AC-600-4-700 at 0 °C and 25 °C is shown in Figure 5b. The Q_st_ of N-AC-3-1000 under low CO_2_ absorption was between 27 and 45 kJ/mol, which was compared with other porous carbon materials [9,11,13]. When the CO_2_ coverage was low, the Q_st_ value was higher, which was due to the adsorption in the pores and the interaction with surface non-uniformity. As the CO_2_ adsorption increased, the Q_st_ decreased, indicating an uneven surface of the material. Figure 5c also shows the CO_2_ absorption bar chart of AC-600-4-700 after 10 repeated regeneration runs at 0 °C. Obviously, although the CO_2_ adsorption capacity remained almost unchanged after 10 repetitions under the same conditions, this indicated that AC-4600-700 had high stability and could recover and capture CO_2_.

### 2.3. Electrochemical Studies

**Figure 6:**
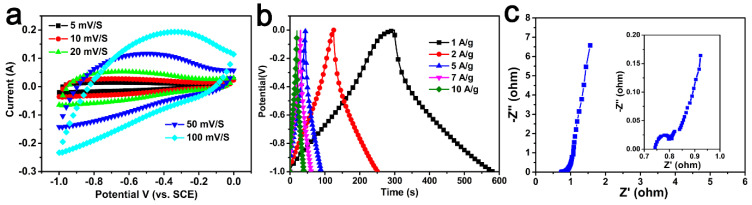
(**a**) CV curves for the AC-600-4-700 electrode sample at different sweep ratios. (**b**) GCD profiles for the AC-600-4-700 at different current densities. (**c**) Nyquist plot of the AC-600-4-700. Inset shown in the high frequencies.

As-obtained carbon materials were also used as electrodes for supercapacitors. Figure 6 and Figure S1 show the CV curves and GCD curves of all of the samples. The specific capacitances of samples were 141–282 F/g, which are shown in Table 1. Among them, AC-600-4-700 had the most superior electrochemical performance. As shown in Figure 6a, it could be observed that the CV curve could maintain a rectangular shape at different scanning rates, indicating that the AC-600-4-700 electrode had excellent electrochemical reversibility [5,44].

Figure 6b shows the charge–discharge curves of the AC-600-4-700 electrode at different current densities. All curves were highly symmetrical with their corresponding charge counterparts in the potential region, indicating pure capacitive behavior. It can be seen that compared to the specific capacitance of 282 F/g at 1 A/g, the specific capacitance at 10 A/g still had 195 F/g, indicating that the electrode had a good discharge rate. These results and analyses confirmed that the AC-600-4-700 electrode exhibited excellent capacitive behavior, which was comparable to most other biomass-derived activated carbons (Table 3) [28,29,30,31,32,33,34,35,36,37,38,39].

The frequency response of the AC-600-4-700 electrode material was investigated by EIS (Figure 6c). The impedance plots show three distinct parts including the high-frequency region, diffusion segment and low-frequency region. The X-intercept of the Nyquist plot at high frequencies showed the equivalent series resistance; a value of 0.64 Ω could be calculated from the illustration. In addition, the slope of about 45° in the low frequency range corresponded to Warburg resistance, which was related to the diffusion resistance of the electrolyte ions entering the electrode material. In addition, the AC-600-4-700 electrode exhibited almost vertical lines in the low frequency range, indicating excellent capacitive behavior [52].

### 2.4. The Relationship between Pore Volume with a Specific Range of Pores and the CO_2_ Adsorption Capacity or Specific Capacity of ACF Samples

**Figure 7:**
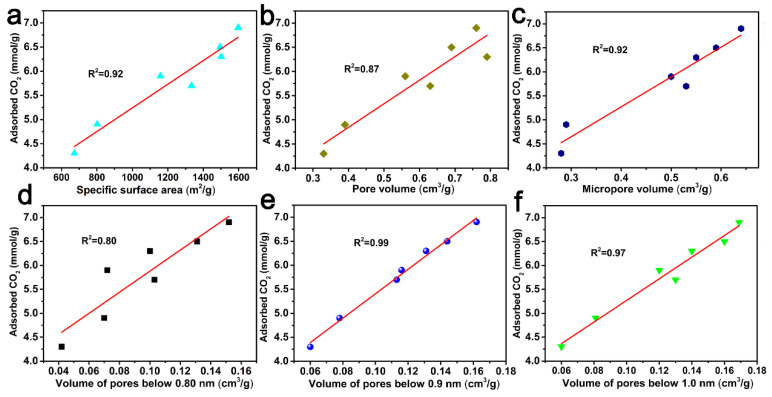
CO_2_ uptake at 0 °C and 1 atm versus specific surface area (**a**), pore volume (**b**), micropore volume (**c**), the pore volume of pores below 0.80 nm (**d**), 0.90 nm (**e**) and 1.0 nm (**f**) derived from CO_2_ adsorption data.

In order to better understand the role of porous structures in the CO_2_ adsorption behavior of ACF samples, the correlation curves between the CO_2_ adsorption capacity and the specific surface area, pore volume, micropore volume, and pore volumes below 0.80 nm, 0.90 nm, and 1.0 nm are shown in Figure 7. And from Figure 7, it can be concluded that pore volumes below 0.90 nm had a better correlation with CO_2_ adsorption at 0 °C and 1.0 atm.

**Figure 8:**
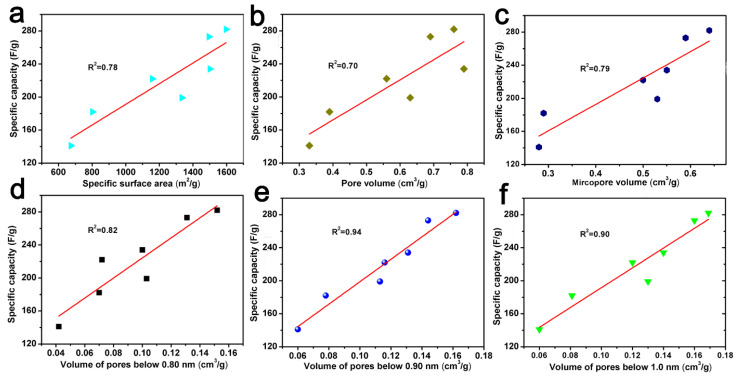
The relationship between specific capacity versus specific surface area (**a**), pore volume (**b**), micropore volume (**c**), the pore volume of pores below 0.80 nm (**d**), 0.90 nm (**e**) and 1.0 nm (**f**) derived from CO_2_ adsorption data.

In addition, the correlation curves between specific capacity and the specific surface area, pore volume, micropore volume, and pore volume below 0.80 nm, 0.90 nm, and 1.0 nm are also shown in Figure 8. It could also show that there is a good correlation between the pore volume and specific capacity below 0.90 nm. This trend seems to indicate that the double layer formed in small pores, i.e., pores with a size of less than 0.90 nm, mainly contributed to the capacitance value [52]. A similar phenomena was also found by Vix-Guterl et al., which indicates that the specific pore volume of pores less than 0.90 nm has an effect on both the CO_2_ capture ability and the electrochemical capacitance [42].

## 3. Materials and Methods

### 3.1. Materials

CF was purchased from the Xinjiang Long staple Cotton Distribution Company, Ürümqi, Xinjiang, China. KOH (>95%) was purchased from Aladdin Chemical Reagent Company, Shanghai, China. Ar gas (>99.9999%) was purchased from Zhongke Kate Co., Ltd., Lanzhou, Gansu, China. All reagents were used without any further purification.

### 3.2. ACFs Material Preparation

CF was firstly carbonized at 500, 600 or 700 °C for 2 h in Ar flow; the resulting carbonized carbon was activated by KOH: a given mass of C-x was impregnated with KOH solutions of different mass ratios (KOH/C-x = 3, 4, and 5), and evaporated under vacuum at 80 °C. Then, the mixture was heated at 600, 700 or 800 °C for 1 h under Ar flow, and the activated sample was neutralized with 1 M HCl until the pH reached 7. The obtained ACF samples were denoted as AC-x-y-z, where x represents the corresponding carbonization temperature, y represents the corresponding KOH/C-x mass ratio, and z represents the corresponding activation temperature.

### 3.3. Characterizations of ACFs Material

A scanning electron microscope (SEM, JSM-6701F, JEOL, Tokyo, Japan) was used to investigate the morphology and microstructure of the prepared samples. The acceleration voltage during the testing process was 0.5–30 kV. Crystallite structures were observed on an XRD (X’ Pert Pro, Philips, Eindhoven, The Netherlands) using Cu Kα radiation from 5° to 90°, a scanning speed of 2 °/min, an acceleration voltage of 45 kV, and a current of 40 mA. An X-ray photoelectron spectroscope (XPS), Physical Electronics, Chanhassen, MN, USA, was used to measure Al Ka radiation. N_2_ adsorption–desorption isotherms were measured at 77 K on a Micrometrics ASAP 2020 volumetric adsorption analyzer (Norcross, GA, USA). Before adsorption measurements were taken, samples were degassed at 200 °C for 4 h. The specific surface area (SSA) and pore size distribution of each sample were calculated from the N_2_ adsorption curve by the BET method and the non-local density functional theory (NLDFT) method, respectively. The total pore volume of the sample was calculated based on the adsorption capacity at a relative pressure of P/P_0_ = 0.99.

### 3.4. CO_2_ Capture Measurement

CO_2_ adsorption isotherms of the ACF samples were measured with a Micromeritics ASAP 2020 static volumetric analyzer at 0 °C or 25 °C between 0.03 and 1 atm. Before each adsorption test, the sample was also degassed at 200 °C for 4 h. The CO_2_ adsorption isotherms were also used to calculate the pore size distribution and pore volume of pores (below 1.0 nm) using the NLDFT model [53]. The N_2_ adsorption isotherm of the ACF sample was measured at 0 °C between 0.03 and 1 atm.

### 3.5. Electrochemical Test

Firstly, the ACF samples were ground into powder. Subsequently, 80 wt% powder active material was mixed with 7.5 wt% acetylene black (>99.9%) and 7.5 wt% conductive graphite in an agate mortar until a uniform black powder was obtained; then 5 wt% poly (tetrafluoroethylene) and a few drops of ethanol were added to the mixture. The resulting paste was coated onto a nickel mesh, dried, pressed at 10 MPa and used as a working electrode.

Electrochemical measurements were performed using a three electrode system in a 2 M KOH electrolyte at room temperature on each prepared electrode using an electrochemical workstation (CHI660D, Shanghai Chenhua Instrument Co., Ltd., Shanghai, China). A Pt electrode and saturated calomel electrode were used as the counter electrode and reference electrode, respectively. Cyclic voltammetry (CV) measurements were performed at different scanning rates, constant current charging/discharging (GCD) measurements were performed at different current densities, and electrochemical impedance spectroscopy (EIS) was studied.

## 4. Conclusions

In this study, we successfully prepared ACF samples with abundant micropores through high-temperature carbonization and KOH activation derived from natural cotton fibers. This study exhibited the highest CO_2_ capture ability of ACF samples at 0 °C and 1 atm, reaching up to 6.9 mmol/g. The ACF samples also showed good selectivity and excellent recyclability for the separation of CO_2_-N_2_. Furthermore, it was found that a specific pore volume below 0.90 nm played a crucial role in determining the CO_2_ capture capacity and electrochemical capacitance. The large capture capacity and high electrochemical capacitance of CO_2_ enable CF to serve as a new biomass source for carbon materials used in CO_2_ capture and high-performance supercapacitors.

## Figures and Tables

**Table 1:** Textural properties of ACF samples derived from CFs.

Sample	SSA (m^2^/g) ^a^	Pore Volume ^a^ (cm^3^/g)	Micropore Volume ^b^ (cm^3^/g)	CO_2_ Uptake 0 °C (mmol/g)	CO_2_ Uptake 25 °C (mmol/g)	Volume of Pores ^c^ ≤1.0 nm (cm^3^/g)	Volume of Pores ^c^ ≤0.90 nm (cm^3^/g)	Volume of Pores ^c^ ≤0.80 (cm^3^/g)	Specific Capacity
AC-500-4-700	673	0.33	0.28	4.3	2.8	0.060	0.060	0.042	141
AC-600-4-700	1597	0.76	0.64	6.9	4.6	0.169	0.162	0.152	282
AC-700-4-700	1158	0.56	0.50	5.9	4.0	0.120	0.116	0.072	222
AC-600-3-700	802	0.39	0.29	4.9	3.6	0.081	0.078	0.070	182
AC-600-5-700	1334	0.63	0.53	5.7	4.0	0.130	0.113	0.103	199
AC-600-4-600	1495	0.69	0.59	6.5	4.4	0.160	0.144	0.131	273
AC-600-4-800	1501	0.79	0.55	6.3	4.2	0.140	0.131	0.100	234

^a^ Data derived from N_2_ adsorption; ^b^ Evaluated by the t-plot method; ^c^ Data derived from CO_2_ adsorption isotherm using the NLDFT model.

**Table 2:** CO_2_ uptakes (at 0 °C and 1 atm) of other biomass-derived activated carbons in comparison with our ACFs ^a^.

Carbon Source	CO_2_ Adsorbed (mmol/g, 0 °C)	CO_2_ Adsorbed (mmol/g, 25 °C)	Ref.
Macadamia nut shell ^b^	6.6	4.4	[8]
Cellulose carbon material from cow dung ^b^	4.6	3.3	[9]
Biochar-based carbon ^b^	6.1	3.7	[10]
Macadamia nut shell ^b^	6.5	4.1	[11]
Petroleum coke ^b^	5.6	3.5	[12]
Coal and rice husk ^b^	6.1	5.0	[13]
Sawdust ^b^	6.6	4.8	[14]
Arundo donax ^b^	6.3	3.6	[15]
Waste surgical mask ^b^	3.9	2.6	[16]
Water hyacinth ^b^	6.0	2.6	[17]
Alligator weed ^b^	6.4	3.4	[18]
Grape marc ^b^	6.7	3.9	[19]
Molasses ^b^	5.2	/	[20]
lignin-based ^b^	5.2	3.6	[21]
Olive stone ^b^	6.1	3.8	[22]
Peanut shells ^b^	5.7	3.7	[23]
Fern leaves ^b^	6.8	3.6	[24]
Spent coffee grounds ^a^	7.2	4.5	[25]
Silk fibers ^a^	7.0	4.8	[27]
Cotton fibers	6.9	4.6	This work

^a^ CO_2_ adsorption capacity at 0 °C and at 1 atm; ^b^ CO_2_ adsorption capacity at 0 °C and 1 bar.

**Table 3:** Biomass-derived activated carbons electrodes and capacitive performance for EDLC.

Carbon Source	Electrolyte	Current Density (A/g)	Specific Capacitance (F/g)	Ref.
Soybean	1 M Na_2_SO_4_	0.1	143	[28]
Cattail	6 M KOH	0.5	127	[29]
Eriocheir sinensis shells	6 M KOH	1	161	[30]
Hybrid willow	6 M KOH	0.1	93	[31]
Bamboo	6 M KOH	1	120	[32]
Tasmanian blue gum tree bark	1 M KOH	1	212	[33]
Tea-waste	6 M KOH	1	332	[34]
Baobab fruit shells	6 M KOH	1	255	[35]
Dandelion flower stem	6 M KOH	0.5	309	[36]
Cotton stalk	1 M H_2_SO_4_	0.2	338	[37]
Rose flowers	6 M KOH	1	350	[38]
Peanut shells	1 M Na_2_SO_4_	1	247	[39]
Cotton Fibers	2 M KOH	1 A/g	282	This work

## Data Availability

Data are contained within the article and Supplementary Materials.

## Supplementary Materials

The following supporting information can be downloaded at: https://www.mdpi.com/article/10.3390/molecules29133153/s1, Figure S1: CV curves for 5 mV·s^−1^ of (**a**) AC-x-4-800 (x = 500, 600 and 700); (**b**) AC-600-y-700, (y = 3, 4 and 5); (**c**) AC-600-4-z (z = 600, 700 and 800); and galvanostatic charge/discharge profiles (GCD) at current density of 1 A·g^−1^ of (**d**) AC-x-4-800 (x = 500, 600 and 700); (**e**) AC-600-y-700, (y = 3, 4 and 5); (**f**) AC-600-4-z (z = 600, 700 and 800).
