# Supplementary material for: Versatile Activated Carbon Fibers Derived from the Cotton Fibers Used as CO2 Solid-State Adsorbents and Electrode Materials
Source: Molecules. 2024 Jul 2;29(13):3153. doi: 10.3390/molecules29133153 (PMC11243228; doi:10.3390/molecules29133153)

Figure S1. CV curves for 5 mV s^{-1} of (a) AC-x-4-800 (x=500, 600 and 700); (b) AC-600-y-700, (y=3, 4 and 5); (c) AC-600-4-z (z=600, 700 and 800); and galvanostatic charge/discharge profiles (GCD) at current density of 1 A g^{-1} of (d) AC-x-4-800 (x=500, 600 and 700); (e) AC-600-y-700, (y=3, 4 and 5); (f) AC-600-4-z (z=600, 700 and 800).

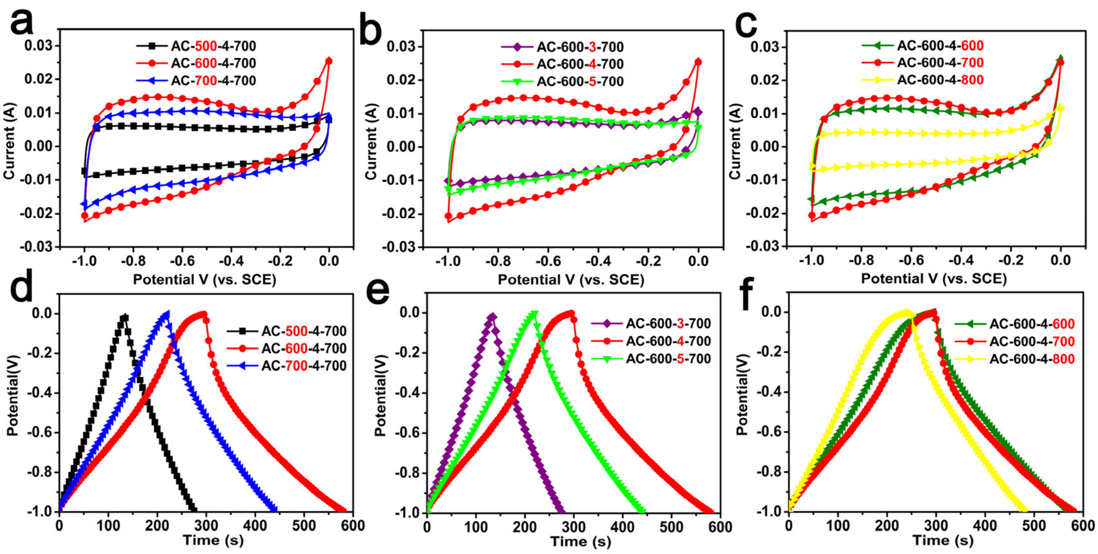

Supplement: Supplementary file 1 [file molecules-29-03153-s001.zip › molecules-3055821-supplementary.pdf]
